# Executive summary: Elite women’s football—Performance, recovery, diet, and health

**DOI:** 10.1111/sms.14145

**Published:** 2022-03-07

**Authors:** Magni Mohr, João Brito, Maysa de Sousa, Svein Arne Pettersen

**Affiliations:** ^1^ Department of Sports Science and Clinical Biomechanics SDU Sport and Health Sciences Cluster (SHSC) University of Southern Denmark Odense Denmark; ^2^ Faculty of Health Centre of Health Sciences University of the Faroe Islands Tórshavn Faroe Islands; ^3^ Portugal Football School Portuguese Football Federation Oeiras Portugal; ^4^ Endocrinology Division School of Medicine Laboratory of Medical Investigation University of São Paulo São Paulo Brazil; ^5^ School of Sport Sciences Faculty of Health Sciences UiT The Arctic University of Norway Tromsø Norway

**Keywords:** competition, female soccer, gender, high intensity exercise, team sport

## Abstract

The present special issue of Scandinavian Journal of Medicine & Science in Sports focuses on performance, recovery, diet, and health in elite women’s football. Beside this summary, an editorial, topic reviews, and original articles written by several of the most published authors in football research are presented. It is, for example, highlighted that there is a great gender inequality in football research in favor of men, especially within elite football populations. Therefore, the broad‐spectrum content of the special issue with focus on several performance areas in women’s football, recovery strategies, nutrition, and psychological factors is highly warranted. Several of the topics examined and data presented are examined for the first on elite women’s football, and therefore, we hope that this special issue will contribute to gender balance the research and emphasis on football in both genders.

## 1 INTRODUCTION

The current special issue of the Scandinavian Journal of Medicine & Science in Sports is fully dedicated to elite women's football. The fifteen articles included aim to provide a comprehensive analysis of several key topics related to elite women´s football and cover areas ranging from muscle physiology to psychology. These areas are associated with performance, recovery, diet, and health in the women´s game.

Overall, the special and interesting history of women´s football is briefly mentioned in the special issue editorial,^1^ and the short‐ and long‐term challenges in the women´s game are discussed. Looking back in history, since the first international game in 1881, there have been periods where women were banned from playing the game. The big picture might be different nowadays, but in science, there is still a clear gender mismatch in research between men´s and women´s football. The gender inequality is elegantly quantified and described in the bibliometric analysis presented by two renown football scientists, Donald Kirkendall and Peter Krustrup.^2^ Their thorough analysis revealed that only a fifth of all football research published is on women, and as low as 15% of the papers on professional football investigated women players. When looking at selected research topics, the numbers are even lower, and, for the first time, scientific evidence is provided on gender inequality in scientific research of football. Thereby, there is a clear need to highly prioritize research in women´s football, and Kirkendall & Krustrup provide a steering instrument showing which areas to focus on in future football research.

The application of invasive field studies has a long tradition in football research, especially in the Scandinavian region. Indeed, already back in 1973, the exercise physiology legend, Bengt Saltin, established an association between performance impairment and muscle glycogen degradation in a male football game.^3^ From this pioneering work and especially during the last three decades, several studies have investigated muscle metabolism and fatigue in football, but entirely in male players. In the current special issue, two papers studied for the first time the muscle metabolic response to a women football game and the importance of muscle characteristics in elite women players.^4^, ^5^ Also, the inter‐individual relationship between skeletal muscle phenotype, physical capacity, and different types of game performance variables were discussed. It was demonstrated that low muscle glycogen and a high number of glycogen depleted muscle fibers is associated with fatigue at the end of a game, and that the skeletal muscle characteristics, such as the sarcolemmal ion transporting system, muscle fiber type, and knee flexor strength, are important variables dictating different types of match‐related performance outcomes. Thus, a muscle physiological link to some of the key match analysis parameters used in modern football is provided in the correlation paper.^5^ Collectively, these two studies^4^, ^5^ pinpoint for some gender differences in elite football, which future research should elucidate in further detail.

Another important area of interest, which also is highlighted in the muscle metabolism paper discussed above,^4^ is that of diet of elite women football players. This topic is covered in an extensive review of the current literature provided by de Sousa and colleagues.^6^ The most pertinent topics for attending physical demands in women players during training and matches were highlighted as being: energy intake, macronutrient and micronutrient requirements, and optimal composition of everyday diet, nutritional and hydration strategies to optimize performance and recovery, potential ergogenic effects of legal, relevant supplements, and future research considerations. In this review, the existing evidence suggests that during the season, a relevant proportion of women football players do not have sufficient energy intake to cope with the energy expenditure during training sessions and games. This may lead to low energy availability that might have detrimental physiological consequences and lead to performance impairment. Furthermore, it has been demonstrated that elite women players often consume low amounts of carbohydrates (3–4 g/kg body mass, an amount that is below the minimum recommended for reaching trainings goals (6–8 g/kg body mass). This review also presents evidence that several ergogenic supplements, namely creatine, caffeine, bicarbonate, beta alanine, and nitrate, may potentially result in improvements in performance and recovery in elite women players. Also, considerations regarding the use of antioxidants and probiotics are discussed.^6^

On the same topic, Oliveira et al^7^ reported the results of a survey exploring the current practices of elite women players regarding the prevalence of dietary supplements, including the types of dietary supplements, the rationale for their use, the sources of information, and the purchase venues. Overall, 82% of the elite players that participated in the study reported using dietary supplements at least once during the previous year, which highlights the need for better and clear information about the potential effects of supplementation. Interestingly, the primary reason for using dietary supplements was to stay healthy, followed by fatigue‐related purposes. These health‐related reasons warrant further investigation, and the scientific evidence of such nutritional options should be discussed with the players. Moreover, during an official international tournament, elite women players did not change the gut microbiota composition over ten consecutive days, even with consecutive training sessions and a congested calendar of matches every 48–72 h.^8^ The report provided by Oliveira et al^8^ also provide novel information for the current knowledge on the gut microbiota in football players, which could be relevant to understand additional factors that modulate athletic performance, recovery, and fatigue.

The women's game presents specific internal and external load demands that need to be elucidated. In the current special issue, Panduro et al^9^ provide position‐specific outcomes and heart rate responses in elite players, with special focus on peak‐intensity periods, elements that were reported in male football nearly two decades ago.^10^ In the Panduro et al study,^9^ the games were shown to be intense and impose high physical demands on all outfield players, with high aerobic loading and relevant declines in high‐intensity activities toward the end of games. Similarly, Winther et al^11^ discussed potential implications for planning of training contents in elite women's football, because during games, transient short peak periods with high amounts of high‐intensity actions, including sprinting, accelerations, and decelerations, occur for several playing positions during match play. This means that individualized, position‐specific conditioning programs, and physical preparation are warranted in elite women's football. With regard to the recovery pattern, Póvoas et al^12^ observed that four consecutive games separated by 48–72 h did not result in considerable alterations in plasma stress markers, physical loading, and technical performance in elite women football players during an official international tournament. Although, more information is warranted about congested fixtures effects on women's football performance and health, the increasing demands of domestic and international competitions worldwide^13^ may impose new challenges for players, coaches, and health and performance staff working in women's football. Therefore, the use of monitoring systems in women elite players such as heart rate variability, as in one of the studies in the present special issue, is of great importance.^14^

It is also noteworthy that in elite women's football, there has been a paucity in research about the importance of strength‐related derivatives on match performance. For the first time, Pedersen et al^15^ investigated potential associations between sprint, jump and squat testing and physical performance as well as peak speed during match‐play. Notably, this study indicated a relationship between 15‐m sprint and accelerations in a game, and a similar pattern between countermovement jump performance and peak speed attained during a game. Thus, new light is shed on the importance of explosive strength in women´s football.

Another area of interest is the psychology of women´s football, which has also been given little attention in the literature.^2^ Pettersen et al^16^ found only 14 papers that met the rather wide inclusion criteria for their review. The most prominent findings displayed that players at higher competitive standard had higher scores on psychological factors such as executive functions, conscientiousness, and mental toughness. However, the cross‐sectional and observational research designs of the studies included limit assumptions about whether these psychologic factors are inherent skills or if they are developed throughout the playing career. Madsen et al^16^ substantiated that age and experience in the sport, as well as national team experience are factors that negatively predicted trait anxiety prior to games. Given the fact that several women players may display high states of anxiety prior to a game, the importance of addressing such issues is warranted, and support should be provided to develop women players’ coping skills. Although, further well‐designed longitudinal studies are warranted on the psychology of female players, the field would also benefit from exploring sound methods for measuring psychological performance objectively.

Overall, it is clear that at the current state of development in women's football, the joining responsibilities between international and national football governing bodies, funding organizations and the research community are warranted. The mismatch in football research done on men and women is too large and has to be addressed in planning of future research strategies. The articles included in the special issue give a broad overview of research in women´s football, as well on important areas such as nutrition, match‐performance, and psychology related to the women´s game. Moreover, completely new areas are such as muscle physiology during an elite women football game are examined for the first time. Therefore, we sincerely hope that this special issue of the Scandinavian Journal of Medicine & Science in Sports fully dedicated to women´s elite football will contribute to a higher understanding of the game of football and for bridging the gap on the gender inequality in football research.

## Data Availability

The data that support the findings of this study are available on request from the corresponding author. The data are not publicly available due to privacy or ethical restrictions.
